# The comparative genomic landscape of adaptive radiation in crater lake cichlid fishes

**DOI:** 10.1111/mec.15774

**Published:** 2021-01-06

**Authors:** Peiwen Xiong, C. Darrin Hulsey, Carmelo Fruciano, Wai Y. Wong, Alexander Nater, Andreas F. Kautt, Oleg Simakov, Martin Pippel, Shigehiro Kuraku, Axel Meyer, Paolo Franchini

**Affiliations:** ^1^ Department of Biology University of Konstanz Konstanz Germany; ^2^ School of Biology and Environmental Science University College Dublin Dublin Ireland; ^3^ National Research Council (CNR) – IRBIM Messina Italy; ^4^ Department of Molecular Evolution and Development University of Vienna Vienna Austria; ^5^ Department of Organismic and Evolutionary Biology Harvard University Cambridge MA USA; ^6^ Max Planck Institute of Molecular Cell Biology and Genetics Dresden Germany; ^7^ Laboratory for Phyloinformatics RIKEN Center for Biosystems Dynamics Research (BDR) Kobe Japan

**Keywords:** adaptive radiation, comparative genomics, demographic history, ecological opportunity, speciation

## Abstract

Factors ranging from ecological opportunity to genome composition might explain why only some lineages form adaptive radiations. While being rare, particular systems can provide natural experiments within an identical ecological setting where species numbers and phenotypic divergence in two closely related lineages are notably different. We investigated one such natural experiment using two de novo assembled and 40 resequenced genomes and asked why two closely related Neotropical cichlid fish lineages, the *Amphilophus citrinellus* species complex (Midas cichlids; radiating) and *Archocentrus centrarchus* (Flyer cichlid; nonradiating), have resulted in such disparate evolutionary outcomes. Although both lineages inhabit many of the same Nicaraguan lakes, whole‐genome inferred demography suggests that priority effects are not likely to be the cause of the dissimilarities. Also, genome‐wide levels of selection, transposable element dynamics, gene family expansion, major chromosomal rearrangements and the number of genes under positive selection were not markedly different between the two lineages. To more finely investigate particular subsets of the genome that have undergone adaptive divergence in Midas cichlids, we also examined if there was evidence for ‘molecular pre‐adaptation’ in regions identified by QTL mapping of repeatedly diverging adaptive traits. Although most of our analyses failed to pinpoint substantial genomic differences, we did identify functional categories containing many genes under positive selection that provide candidates for future studies on the propensity of Midas cichlids to radiate. Our results point to a disproportionate role of local, rather than genome‐wide factors underlying the propensity for these cichlid fishes to adaptively radiate.

## 1 INTRODUCTION

Only some lineages of organisms phenotypically diversify and rapidly speciate to form adaptive radiations. Many others do not. The interplay between mechanisms at different levels of biological complexity ranging from ecology to genomics that could generate these disparate patterns of organismal diversification is increasingly amenable to analyses (Franchini et al., 2019; Schluter et al., 2010; Whitehead, 2012; Xiong et al., 2018). Adaptive radiations can be promoted by both extrinsic and intrinsic factors. One such extrinsic factor is ecological opportunity whereby the first lineage that is able to invade a novel habitat such as an island or lake that lacks competing taxa might have an advantage (through a priority effect) that favours further diversification (Schluter, 2000; Simpson, 1953; Stroud & Losos, 2016). However, priority effects and resulting initial advantages are not likely to be the only determinants of the likelihood of forming adaptive radiations (Losos, 2010). For instance, the probability that a lineage diversifies could be greatly increased if particular genomic factors primed a lineage to adaptive divergence (Brawand et al., 2014; Franchini et al., 2019; Karagic et al., 2020; Svardal et al., 2020). A wide array of genome‐wide changes ranging from chromosomal duplications, to expansions of gene families, to bursts of transposable elements (TEs) could predispose particular lineages to radiate adaptively (Brawand et al., 2014; Faber‐Hammond et al., 2019; Feiner, 2016). Many of these intrinsic genomic factors may also not alter the entire genome, but could instead represent modifications at particular genomic regions that repeatedly underlie certain adaptations or even particular gene pathways that enhance the probability of subsequent diversification (Kratochwil et al., 2018; Miller et al., 2014; Morris et al., 2019). To investigate the potential contributions of these extrinsic and intrinsic mechanisms in the formation of adaptive radiation in Nicaraguan lakes, we examined the population and comparative genomics of two closely related lineages of cichlid fishes, the adaptively radiating Midas cichlids and the nonradiating lineage of *Archocentrus centrarchus*.

Midas cichlid fishes (*Amphilophus citrinellus* species complex) represent a model system for genomic studies of adaptive radiation in sympatry (Barluenga et al., 2006; Elmer et al., 2010; Franchini et al., 2019). Together with several other adaptive radiations, cichlid fishes comprise more than 2,000 species that exhibit unparalleled phenotypic diversity and dominate the fish communities of many tropical freshwater environments in Africa and the Neotropics (Lopez‐Fernandez et al., 2010; Seehausen, 2006; Verheyen et al., 2003). In Central America, Midas cichlids colonized a chain of crater lakes from two large source lakes, Lake Nicaragua and Lake Managua, <5,000 years ago (Kautt et al., 2020). This lineage then diversified into at least 13 species, some of which have evolved within the last 2,000 years, following the formation of these crater lakes (Elmer et al., 2010; Kutterolf et al., 2007). In one of these crater lakes, Lake Xiloá, *Am. citrinellus* has diversified into at least four species, the benthic high‐bodied species *Am. amarillo*, *Am. xiloaensis* and *Am. viridis*, as well as the limnetic arrow‐shaped species *Am. sagittae*. Compared to the large but shallow source lakes, these recently formed Nicaraguan crater lakes are small but deep, allowing phenotypic divergence along the benthic–limnetic axis (Recknagel et al., 2013; Stauffer & McKaye, 2002). Indeed, Midas cichlids in these crater lakes show substantial diversity in traits associated with this axis, including body and pharyngeal jaw morphology. The body shape is more elongated in open‐water species and is associated with divergence in swimming performance (Elmer, Kusche, et al., 2010; Franchini et al., 2014; Raffini et al., 2020). Additionally, pharyngeal jaws tend to be more enlarged in bottom‐dwelling species – providing better performance at processing hard food items (Barluenga et al., 2006; Fruciano, Franchini, Kovacova, et al., 2016; Meyer, 1989, 1990). The habitat stratification in these crater lakes, coupled with putatively competitor‐free environments, might have repeatedly provided novel ecological opportunities for these Midas cichlids to diversify in the crater lake habitats, and has been suggested to be a main factor facilitating their radiation (Kautt et al., 2018; Recknagel et al., 2014). If Midas cichlids did invade these crater lakes before other closely related fishes, phenotypic traits contributing to their success could have diverged without intrinsic genomic factors playing a significant role. Importantly, several cichlid lineages other than the Midas cichlid species flock also live in these crater lakes. However, there is no evidence that any of these other lineages have speciated within this system (Elmer et al., 2013; Franchini et al., 2017; Fruciano et al., 2016) and they show little phenotypic divergence (Fruciano, Franchini, Kovacova, et al., 2016; Fruciano, Franchini, Raffini, et al., 2016). These closely related cichlid lineages, such as the flyer cichlid *Ar. centrarchus* that shared a last common ancestor only a few million years ago with the Midas cichlid lineages (Hulsey et al., 2010), provide ideal candidates for comparisons with Midas cichlids since they also live in the same habitats including the older large Lakes Nicaragua and Managua as well as several of the much younger crater lakes.

Both ecological opportunity and several intrinsic factors linked to genomic architecture could have promoted diversification in Midas cichlids and not in *Ar. centrarchus*. For instance, there are a number of genome‐wide changes that can be evaluated following the construction of reference genomes and that could be identified as factors promoting the adaptive radiation of Midas cichlids. Such genome‐wide changes might include the duplication of entire genomes as has been implicated in the radiations of many plant lineages and virtually all teleost fishes (Glasauer & Neuhauss, 2014; Sandve et al., 2008; Wittbrodt et al., 1998). The duplication or fusion of individual chromosomes could also favour speciation and lineage diversification as has been suggested in groups as diverse as the Hawaiian silverswords, sunflowers, notothenioid fishes and mice (Carr & Kyhos, 1986; Faria & Navarro, 2010; Franchini et al., 2020; Rieseberg, 2001). The increase in genome size associated with any novel genetic material might also be accompanied by a pronounced increase in members of gene families providing the raw material for the rapid evolution of novel gene functions (Ohno, 1970). For instance, the expansion of anti‐freeze protein copy numbers in Antarctic fishes has long been recognized as key to their adaptive radiation in cold‐water environments (Baalsrud et al., 2018). Copy number variation has also been identified to differ substantially among lake radiations and even closely related species among African cichlid lineages (Faber‐Hammond et al., 2019). Further, the Midas cichlids could have also experienced an increase of TEs in their recent past that might have favoured extensive mutability of the genome and generated rapid changes in processes such as gene regulation as has been implicated in the radiations of Anolis lizards as well as East African rift lake cichlids (Brawand et al., 2014; Feiner, 2016). A disproportionate number of new chromosomes, gene families or TEs could have readily facilitated adaptive diversification in Midas cichlids as compared to other cichlids found in similar ecological contexts. Comparisons of entire high‐quality genome assemblies make these types of assessments between lineages nowadays possible.

Islands of localized genomic divergence are also receiving increased scrutiny in adaptive radiations ranging from Heliconius butterflies to Hawaiian honeycreepers (Campana et al., 2020; Morris et al., 2019). Apart from genome‐wide events, processes affecting local genomic regions, for instance, adaptive loci of genes with large phenotypic effects, might also have facilitated adaptive divergence in Midas cichlids. Several quantitative trait loci (QTL) have been identified in particular lineages of Midas cichlids that are associated with variation in body and pharyngeal jaw shape (Fruciano, Franchini, Kovacova, et al., 2016). It seems reasonable that such regions contributing to local adaptation would show marked differences in their TE content, number of genes under selection or other types of genomic divergence (Bohne et al., 2008; Brawand et al., 2014). QTL responsible for species divergence could also include multiple loci spanning substantial components of the genome that influence the same or even multiple adaptively diverging traits (Erickson et al., 2018; Nelson et al., 2019; Pardo‐Diaz & Jiggins, 2014; Shahandeh & Turner, 2020). Midas cichlids have diverged in parallel along the limnetic‐benthic axis in crater Lake Apoyo and crater Lake Xiloá in multiple traits along strikingly similar phenotypic trajectories (Elmer et al., 2014). Both limnetic species differ phenotypically from the benthic species in having a more elongated body as well as in the shape and dentition of their pharyngeal jaws. This divergence between these two crater lake radiations has likely also occurred over a very short timeframe (~5,000 years). Similarly, parallel divergence in traits such as pelvic spines and armoured plates in multiple freshwater lineages of three‐spine stickleback that last shared a common ancestor during the last glaciation has often been suggested to be due to divergence at similar loci and in some cases due to the same alleles (Miller et al., 2014; Schluter et al., 2010). These populations are often separated by hundreds of kilometres. Further, in cichlid radiations in both lakes Malawi and Victoria in east Africa that are also separated by several hundreds of kilometers, traits like stripes and tooth numbers also show divergence in the same QTL (Hulsey et al., 2017; Kratochwil et al., 2018). Given the large extent of phenotypic parallelism in the two Nicaraguan crater lakes that are geographically separated by only about 100 km (Elmer et al., 2014), it is reasonable to assume that their repeatedly diverging traits could share, at least partially, the same genetic basis. Under this assumption and given that a QTL study in Lake Xiloá is not yet available, we focus our analysis on the QTL regions detected in a Lake Apoyo benthic–limnetic cross. Comparative genomics should allow us to discount a number of genomic characteristics that do not differ and identify previously unidentified differences between the nonradiating lineage of Nicaraguan cichlids and the adaptive radiation of Midas cichlids that might have affected their propensity to form adaptive radiations.

To examine the genomic divergence characterizing the adaptively radiating Midas cichlid fishes, we examined the similarities and differences in the genomic composition of both the Midas cichlid *Amphilophus citrinellus* and its nonradiating relative *Archocentrus centrarchus*. We first generated high‐quality genome assemblies for both species. Then, whole‐genome resequenced population data were used to reconstruct the demographic history of *Ar. centrarchus* that colonized crater Lake Xiloá from the larger Lake Managua and contrast it with co‐occurring members of the Midas cichlid species complex. These two lakes were chosen because Lake Managua is considered the source lake for the colonization of Lake Xiloá for both lineages and because in Lake Xiloá there is evidence of adaptive radiation in Midas cichlids, but not in *Ar. centrarchus* (Franchini et al., 2017; Fruciano, Franchini, Raffini, et al., 2016). The reference genomes were then examined for genome size, gene sequence evolution, expansion of gene families and whole‐genome TE content to evaluate whether there are any large‐scale differences between the nonradiating and the radiating lineage. Additionally, we examined patterns of divergence between the two genomes in several local regions containing QTL for phenotypic divergence in Midas cichlids found in crater Lake Apoyo. Finally, we identified genes that show evidence of adaptive molecular evolution and determined if these genes were clustered in particular gene pathways. Together, these analyses provide an integrative and comparative examination of the genomic conditions underlying the Midas cichlid adaptive radiation.

## 2 METHODS

### 2.1 Reference genome sequencing and de novo genome assembly

To generate the reference genomes, high molecular weight DNA from muscle and fin tissues was extracted from a single adult female of each of the two species *Amphilophus citrinellus* and *Archocentrus centrarchus* using the MagAttract HMW DNA Kit (Qiagen). For *Am. citrinellus*, paired‐end sequencing libraries were constructed using the TruSeq DNA HT Library Prep Kit (Illumina) with three different insert sizes (300, 500 and 700 bp), while the Nextera Mate Pair Library Prep Kit (Illumina) was used to build long‐insert mate‐pair libraries (3,000 and 5,000 bp). Additionally, we generated long‐jump libraries with insert sizes of up to 22 Kb using a proprietary protocol at Eurofins. For *Ar. centrarchus*, paired‐end libraries were constructed using the NEBNext Ultra II DNA Library Prep Kit for Illumina (New England Biolabs) with four different insert sizes (300, 500, 700 and 900 bp) and a mate‐pair library with a mate distance of 7–10 Kb was prepared using 4 µg of genomic DNA with the Nextera Mate Pair Library Preparation Kit (Illumina) following the customized protocol iMate (Tatsumi et al., 2015). DNA quality and quantity were assessed using a Bioanalyzer 2100 (Agilent Technologies) and a qubit v2.0 fluorometer (Life Technologies). Sequencing was carried out at the University of Konstanz Genomics Center (GeCKo), the Tufts University genomics facilities (TUCF Genomics), Laboratory for Phyloinformatics of RIKEN Kobe Campus and the University of Edinburgh (Edinburgh Genomics) (see Table S1 for platforms used and sequencing statistics).

For *Am. citrinellus*, read pairs were generated via in vitro proximity ligation, performed by an external service provider using a proprietary protocol (Dovetail Genomics). Dovetail ‘Chicago’ libraries were prepared as described in Putnam et al. (2016) from flash‐frozen liver tissue. Genomic DNA was reconstituted into chromatin in vitro and fixed with formaldehyde. Fixed chromatin was digested with DpnII and 5′‐GATC overhangs were left. The 5′ overhangs were marked with biotin‐14‐dATP, and then, the free blunt ends were ligated. After ligation, the DNA was purified by reversion of crosslinked proteins and incubation with proteinase‐K. The un‐ligated biotin was removed from the purified DNA. Afterwards, the DNA was sheared to an average fragment size of 350 bp and a single library was generated using NEBNext Ultra enzymes and Illumina‐compatible adapters and sequenced at TUCF Genomics.

For *Ar. centrarchus*, Bionano Genomics optical maps were generated with a Saphyr genome mapping system at the Queen Mary University Genomics facility (London, UK). Extraction of megabase genomic DNA for Bionano optical mapping was done according to the IrysPrep Animal Tissue protocol (bionano tech note v. 1.1.12). Briefly, cell nuclei were isolated from *Ar. centrarchus* liver tissue and embedded in agarose plugs. After proteinase K and RNAse treatment of plugs, genomic DNA was extracted and cleaned by drop dialysis against 1× TE buffer. PFGE revealed DNA molecules with a minimum of 100 Kb and up to 1 Mb of length. The purified DNA sequence‐specific labelling was performed by the Nick, Labelling, Repair and Staining steps according to IrysPrep TM NLRS. Sequence specificity was provided by the nickase nt.bspq1. Labelling was carried out by a nick translation process in the presence of a fluorophore‐labelled nucleotide. The labelled nicks were repaired to restore strand integrity, and DNA molecules were stained for visualization. The molecules were imaged using the Irys system that loads stained molecules automatically into Bionano Genomics nanochannel chips using electrophoresis. Label positions and lengths of DNA molecules were recorded by the on‐board CCD camera using green and blue lasers in the Bionano Genomics Irys system. Data were generated from four flow cells.

The program trimmomatic v0.36 (Bolger et al., 2014) was used to remove adapters and to filter the reads by quality with default settings and to discard sequences shorter than 50 nucleotides. Given the high heterozygosity of the species (we used a wild‐caught individual for *Am. citrinellus* and an F1 individual from wild‐caught fish for *Ar. centrarchus*), we employed the genome assembler Platanus, an effective tool for assembling highly heterozygous genome sequences (Kajitani et al., 2014). For each species, quality‐filtered reads were assembled with platanus 1.2.4 (Kajitani et al., 2014) by applying three main steps: (a) contigs were assembled using paired‐end reads, with a 67‐mer extension to construct de Bruijn graphs; (b) contigs were joined together in a scaffolding step using both paired‐end and mate‐pair information; (c) scaffolds were processed using a gap‐closing step to minimize unknown bases. For *Am. citrinellus*, the proximity ligation technology of Dovetail Genomics was then applied to further improve scaffolding. To this end, read pairs generated with the ‘Chicago’ protocol were aligned to the Platanus scaffolds and processed by Dovetail's HiRise assembly algorithm (Putnam et al., 2016). This allowed us to produce a likelihood model for the genomic distance between read pairs, and the model was used to identify and break putative misjoins, to score prospective joins, and to make joins above a threshold. A final set of sequences, including the unplaced and the HiRise‐scaffolded sequences, was output in fasta format.

To further scaffold the assembled genome of *Ar. centrarchus*, we relied on a high‐coverage Bionano Genomics optical map that was assembled de novo and used to order and orient the scaffolds from the Platanus pipeline and to correct misassemblies. Consensus physical maps (CMAPs) were assembled using bionano solve 3.2.1. Molecules from four flow cells were merged, and a signal‐to‐noise ratio (SNR) filtering was applied. Subsequently, molecules were filtered for a minimum length of 100 Kb and a minimum of eight labels on each molecule (n = 4,190,419; approximately 736X raw coverage). A *p*‐value threshold for the optical mapping assembly was set to at least 1 × 10^−10^. A total of 2,775 CMAPs (N50 of 0.769 Mb; total CMAP length of 1,782,764 Mb) were generated. We then used the bionano solve 3.2.1 hybrid‐scaffolding pipeline with input parameters optimized for human. In short, the process of hybrid scaffolding includes alignment of the Illumina assembly against the Bionano physical maps, identifying and resolving conflicting alignments, merging of nonconflicting assembly and CMAPs into hybrid scaffolds, and the final translation back to fasta format. As a final step to correct remaining base errors, we aligned all the short reads of each species to the corresponding genome assembly with bwa‐mem v0.7.15 (Li & Durbin, 2009), and we used freebayes v1.1.0 to detect polymorphic positions and fix erroneous nonpolymorphic sites in the reference sequence with bcftools consensus (Li et al., 2009). Genome completeness was assessed with busco v3 (Waterhouse et al., 2018) using the Core Vertebrate Genes (CVG) in the gVolante webserver (Nishimura et al., 2017).

### 2.2 Genome size estimation

The filtered high‐quality paired‐end Illumina short‐reads (see paragraph ‘Sample preparation, sequencing and de novo genome assembly’) were used to estimate the genome size of *Amphilophus citrinellus* and *Archocentrus centrarchus* based on k‐mer frequency (k‐mer value: 21, 25 and 29). To this end, the program jellyfish v2.2.6 (Marcais & Kingsford, 2011) was used to count the occurrence of each k‐mer in the sequence set of each species. The jellyfish output was then processed by genomescope v1.0 (Vurture et al., 2017) to estimate genome heterozygosity, repeat content and size using a k‐mer‐based statistical approach. Shape and size of the k‐mer graph are modelled by the algorithm implemented in GenomeScope using four negative binomial peaks and determined by the rate of heterozygosity, PCR duplication and PCR error.

### 2.3 Transcriptome sequencing and assembly

For this project, we used both publicly available and newly generated RNA‐Seq data to annotate the protein‐coding loci in the two genomes (see Table S2). For *Am. citrinellus*, a compilation of RNA‐Seq samples including various developmental stages (1 day posthatch, 1 and 3 months posthatch and adult) and tissues (whole body, eyes, lips, pharyngeal jaws, skin) was used. For *Ar. centrarchus*, 1‐day post‐hatch embryos and brain, liver, gonads, pharyngeal jaw, skin, muscle and spleen from one adult individual were processed. Total RNA was isolated using a Promega ReliaPrep miRNA tissue kit according to the manufacturer's instructions (Promega). From approximately 50 ng of total RNA per sample, sequencing libraries were constructed using either the ‘SENSE mRNA‐Seq Library Prep Kit’ (Lexogen) or the TruSeq Stranded mRNA Library Prep (Illumina) according to the manufacturer's recommendations. A qubit v 2.0 and a bioanalyzer 2100 were used in the different quality and quantification steps. Paired‐end sequencing (2 × 151 bp) was conducted either in Illumina HiSeq2500 platforms at the genomics facility of the Tufts University of Boston (TUCF Genomics) or in an Illumina HiSeq X‐Ten at BGI, the Beijing Genomics Institute (see Table S2 for sequencing statistics).

Raw reads were filtered using trimmomatic v0.36 (see the previous paragraph) and used to assemble the *Am. citrinellus* and *Ar. centrarchus* transcriptomes. For each species independently, we used the program trinity v2.8.4 (Haas et al., 2013) to first generate a de novo transcriptome assembly and then second a reference‐guided assembly. In both approaches, Trinity was run with default settings and sequences shorter than 200 bp were discarded. For the reference‐guided Trinity runs, reads from each species were aligned to the corresponding genome assembly using the splice‐aware program hisat2 v2.1.0 (Kim et al., 2015) with default settings. The completeness of the combined de novo and reference‐guided set of transcripts was evaluated with busco v3 (CVG database) in gVolante.

### 2.4 Annotation of protein‐coding genes

To create gene models for *Am. citrinellus* and *Ar. centrarchus*, we used an evidence‐based gene prediction approach that relied on the program evidencemodeler (EVM) v1.1.1 (Haas et al., 2008). Following this approach, we combined ab initio gene prediction, homology‐based prediction and transcript reconstruction into weighted consensus gene structures (see Figure S1 for a schematic overview of the annotation pipeline). Ab initio prediction: RNA‐seq data were generated from different tissues and developmental stages for the two species (Table S2). Quality‐filtered reads from each species were aligned to the corresponding genome using the splicing‐aware mapping program hisat2 with default settings. The pipeline braker v2.0 (Hoff et al., 2016), which combines the advantages of the implemented software genemark‐et v4.57 (Tang et al., 2015) and augustus v3.3 (Stanke et al., 2006), was run on mapped files and the soft‐masked version of the two genomes (see the previous paragraph). Briefly, genemark‐et is first used to carry out iterative training and generate initial gene structures, and then, Augustus uses predicted genes for training and finally integrates the RNA‐seq mapping information into a more comprehensive gene prediction. Homology‐based prediction: Protein sequences from four fish species (Nile tilapia, *Oreochromis niloticus*; three‐spined stickleback, *Gasterosteus aculeatus*; zebrafish, *Danio rerio*; spotted gar, *Lepisosteus oculatus*), chicken (*Gallus gallus*), mouse (*Mus musculus*) and human (*Homo sapiens*) were retrieved from the Ensembl database release 91. Additionally, manually curated fish proteins were downloaded from the UniProtKB/Swiss‐Prot release 2019_03 database (The UniProt Consortium, 2019). The whole protein data set, including 175,421 entries, was aligned to the repeat‐masked *Am. citrinellus* and *Ar. centrarchus* genomes using exonerate v2.2.0 (Slater & Birney, 2005) with default settings. Transcript reconstruction: For each species, transcripts were reconstructed using the pasa pipeline v2.2.3 (Haas et al., 2003) independently. The combined de novo or the reference‐guided transcriptome assembled transcripts were first processed by the seqclean tool (https://sourceforge.net/projects/seqclean) to remove poly‐A tails and other contaminant sequences. To obtain nonredundant alignment assemblies, PASA was run with the options ‘—TRANSDECODER; ‐‐MAX_INTRON_LENGTH 1000000’ and using as transcript aligner the programs gmap v2019‐05‐12 (Wu & Watanabe, 2005) and blat v36x2 (Kent, 2002). As additional transcript‐based evidence, transcripts were reconstructed with stringtie v1.3.6 (Pertea et al., 2016) by parsing the alignments produced by hisat2. The Stringtie‐reconstructed transcripts were processed by the program transdecoder v5.5.0 (https://github.com/TransDecoder) to infer potential open reading frames (ORFs) in each assembled transcript (TransDecoder.LongOrfs) that are at least 100 amino acids long, predict likely coding regions (TransDecoder.Predict) and finally generate a genome‐based coding region annotation file.

EVM was then used to create a consensus gene model from the obtained gene evidence by setting the following weights: (pasa assemblies: 10; exonerate: 5; augustus: 1; genemark‐et: 1; stringtie/transdecoder transcripts: 1). The pasa pipeline was then used to update the EVM consensus predictions by adding untranslated region (UTR) annotations and models for alternatively spliced isoforms. Finally, genes with a complete overlap with repeat elements were removed. The full set of transcripts was extracted from the genome and evaluated for completeness using busco v3 (CVG database) in gVolante.

### 2.5 Whole‐genome resequencing and demographic history inference

For the demographic analyses of both *Am. citrinellus* and *Ar. centrarchus*, we resequenced the genomes of 40 individuals (20 per lineage), and we conducted of series of analyses to (a) elucidate the demographic history of the two lineages spanning the colonization of crater lake Xiloá from great lake Managua and (b) to further identify relevant differences in their genomes.

To generate resequenced genome data, HMW DNA was extracted from fin or muscle tissue from 20 Midas cichlids (10 *Am. citrinellus* fishes from Lake Managua and 10 *Am. amarillo* from Lake Xiloá) and 20 *Ar. centrarchus* (10 individuals for each lake) using a Qiagen DNeasy Blood & Tissue kit, including an RNase A treatment step. DNA integrity was manually inspected on agarose gels and concentrations were determined on a qubit v2.0 fluorometer. Genomic libraries were prepared using an Illumina TruSeq DNA Nano kit aiming for 350 bp insert sizes. Genomic libraries were paired‐end sequenced (2 × 150 bp) on HiSeq X‐Ten Illumina platforms at BGI, pooling five individuals per lane.

For demographic inferences, we used whole‐genome resequencing data for the 40 individuals (10 per lake/species combination). We implemented angsd v0.929 to estimate the observed two‐dimensional site‐frequency spectra (2D‐SFS). Applying the GATK model to calculate genotype likelihoods, we only included reads that were mapped in proper pairs and had a minimum mapping quality of 30 and only considered sites with at least eight individuals with ≥5× sequencing coverage after filtering (base quality ≥ 20). Moreover, nonbiallelic SNPs and SNPs exhibiting a significant strand bias or deviation from Hardy–Weinberg equilibrium (*p* < 0.01) were excluded. To polarize SNP alleles for the unfolded 2D‐SFS, we first generated ancestral reference genomes for *Ar. centrarchus* and *Am. citrinellus* by using the *Am. citrinellus* and *Ar. centrarchus* samples, respectively, as outgroup to represent the ancestral SNP states. For this, we used the ‘‐doFasta 2’ option in angsd to generate fasta sequences representing the most common allele at each site. Additionally, we masked polymorphic sites in the outgroup samples (i.e. sites with ambiguous ancestral state) by using angsd with the ‘‐doMaf 2’ option and applying a SNP *p*‐value cut‐off of .05. Using the corresponding ancestral reference genome, we estimated the unfolded 2D‐SFS for the two population/species pairs with the ‘realSFS‘ program of the angsd package.

The expected and observed 2D‐SFS were compared with gadma v1.0.0 (Noskova et al., 2020), using the Powell's conjugate direction method implemented in moments v1.0.2 (Jouganous et al., 2017) and four repeats with default parameters. The examined models were restricted to a structure with two changes each before and after the population split without gene flow. The best fit model was considered as the most plausible evolutionary scenarios for the colonization of Lake Xiloá by each species. Confidence intervals of the maximum‐likelihood parameter estimates were assessed with 100 nonparametric bootstrap replicates by resampling sites in the 2D‐SFS with replacement.

To further investigate the differences in the age of colonization of crater Lake Xiloá between the two lineages, we compared a series of models by fitting simulated 2D‐SFSs to the empirical data using fastsimcoal v.2.6 (Excoffier et al., 2013), closely following the strategy used in (Kautt et al., 2020). Briefly, we ran 100 independent fastsimcoal runs from different parameter starting values for each model, optimizing parameters for 100 ECM cycles and estimating the expected site‐frequency spectra using 200,000 coalescent simulations. To obtain confidence intervals around the maximum‐likelihood parameter estimates, we applied a parametric bootstrapping approach: we simulated 100 unfolded 2D‐SFSs with fastsimcoal following the length distribution of the corresponding reference genome and using demographic parameters from the run with the highest likelihood. For each simulated SFS, we performed 10 independent optimization runs using 40 ECM cycles and 200,000 coalescent simulations.

### 2.6 Signatures of selection from whole‐genome resequencing data

To investigate signatures of balancing and directional selection in the source lake populations of the radiating and nonradiating lineages, we used the genotype‐free approach implemented in angsd to calculate theta π and Tajima's D statistics across both reference genomes. We applied the same filters as for generating the 2D‐SFS but used the folded 1D‐SFS as priors for each population for calculating site‐wise summary statistics with the ‘realSFS’ program of the angsd package. Using the site‐wise estimates, we applied the ‘thetaStat’ program of the angsd package to calculate summary statistics in nonoverlapping 10‐kb windows.

Standing genetic variation has long been recognized as an important factor to promote rapid adaptation, as alleles segregating in a population can be recruited to mediate adaptive responses in novel environments, hence circumventing the need to wait for the emergence of new mutations (Barrett & Schluter, 2008). The extent of standing genetic variation available for adaptive responses in the novel crater lake environment depends on the evolutionary histories of the lineages in the source lakes, such as ancestral and founder population sizes, mutation rates, strength of background selection and the occurrence of directional and balancing selection affecting loci underlying the adaptive response. To evaluate the levels of standing genetic variation at loci potentially involved in adaptive diversification, we compared the distributions of theta π and Tajima's D statistics between windows within and outside of QTL regions for the populations of both lineages in Lake Managua. Theta π is an estimator of genetic diversity, while Tajima's D is informative about the past selective history when comparing different genomic regions. In both cases, larger values of these statistics in QTL regions as compared to the genomic background would be indicative of the presence of clusters of old and divergent haplotypes segregating in the source population, while lower values would hint at recurrent selective sweeps or strong effects of background selection at these loci. We argue that the presence of high levels of standing genetic variation at loci underlying key adaptive traits might provide the substrate for strong divergent selection among ecotypes in the crater lakes, which would facilitate rapid lineage diversification in the crater lakes.

### 2.7 Transposable elements (TEs) annotation and analysis

Transposable elements in *Am. citrinellus* and ten *Ar. centrarchus* were identified de novo with repeatmodeller v1.0.0 (Smit & Hubley, 2008‐2015) and repeatmasker v4.0.7 (Smit et al., 2013‐2015). repeatmasker was run with the species‐specific library, as well as the master repeatmasker combined library (Dfam_Consensus RELEASE 20170127 and RepBase RELEASE 20170127). The repeatmasker output file was parsed and the ‘perc.div’ information was used for the sequence divergence plot. The Euclidean distance matrix of TE genome composition of each TE class (i.e. total base pair of TE class/genome size) was calculated in R using the *stats* package. The networks were visualized using cytoscape v3.7.2 (Shannon et al., 2003).

### 2.8 Synteny analysis

Pairwise orthologous genes between *Am. citrinellus* and *Ar. centrarchus* were identified by orthofinder v2.0.0 (Emms & Kelly, 2019) using default settings. Orthologous information and gene annotations were parsed and used as input files in the custom macro‐synteny pipeline. Scaffolds in both species were clustered and reordered based on the number of shared orthologous genes. Only scaffolds with more than 10 orthologous genes identified by Orthofinder were kept, and orthologous genes with more than 4 copies in the genome were filtered. To study the synteny and collinearity, putative homologous chromosomal regions between *Am. citrinellus* and *Ar. centrarchus* were identified using MCScanx (Wang et al., 2012). The all‐to‐all blast input for MCScanx was produced using blast+v2.2.31+ (Altschul et al., 1990) with an E‐value cut‐off of 1e^−7^.

### 2.9 Positive selection on protein‐coding genes and gene family evolution

The annotated genomes of five African cichlid fishes (*Maylandia zebra*, *Pundamilia nyererei*, *Astatotilapia burtoni*, *Neolamprologus brichardi*, *Oreochromis niloticus*) and three noncichlid teleost fishes (*Oryzias latipes*, *Xiphophorus maculatus*, *Danio rerio*) were downloaded from the Ensembl 98 database (Cunningham et al., 2019). These eight fishes were included in the comparative analyses aimed at investigating genomic differences between *Am. citrinellus* and *Ar. centrarchus*. To detect orthologous relationship among these genomes, the longest transcript for each gene was selected as a canonical transcript to represent that gene. All‐against‐all blast (blastp v2.2.31+) (Altschul et al., 1990) was employed on the protein sequences of these canonical transcripts with an E‐value cut‐off of 1e‐7. The high‐scoring segment pairs (HSPs) of the blast result were parsed and conjoined using the program solar v0.9.6 (Yu et al., 2006). The similarity between protein sequences across the combined ten species was evaluated based on bit‐score from blast results. Protein sequences were then clustered into gene families using a hierarchical clustering algorithm (hcluster_sg v0.5.1) implemented in the Treefam pipeline (parameters: ‘‐w 5; ‐s 0.33; ‐m 100000’) (Schreiber et al., 2014).

The phylogeny of these ten species was estimated with raxml v8.2.4 (Stamatakis, 2006) using concatenated protein sequences of 1,788 single‐copy ortholog genes. To calibrate divergence times, the divergence time of the outgroup *D. rerio* was preset to 230 million years (Kumar et al., 2017). Then, the concatenated alignment of fourfold degenerate sites of single‐copy ortholog genes was analysed by the mcmctree program from the paml v4.9 package (Yang, 2007). To test which genes were under positive selection in the two branches of *Am. citrinellus* and *Ar. centrarchus*, the branch‐site model of paml package (Yang, 2007) was used setting the foreground branch as *Am. citrinellus* and *Ar. centrarchus*, respectively. The ratio of nonsynonymous substitutions to synonymous substitutions (dN/dS) was estimated. Likelihood ratio tests (LRT) were performed on the null model (sites under neutral evolution on foreground branches to the background) and alternative model (a proportion of sites under positive evolution on foreground branches) with a 0.05 significant cut‐off. The gene family evolution analysis was performed using cafe v4.2.1. The gene family expansion and contraction were estimated across the phylogeny for 10,957 gene families that contain paralogs.

### 2.10 Linkage map construction and QTL mapping

The linkage map for QTL mapping was generated previously and originally reconstructed de novo (Franchini et al., 2014; Fruciano, Franchini, Kovacova, et al., 2016). Here, we used the genomes to reconstruct positional information of our double digest restriction‐site associated DNA (ddRADseq) from two parental and 306 F_2_ individuals from an *Amphilophus astorquii* × *Amphilophus zaliosus* cross (crater lake Apoyo) (Fruciano, Franchini, Kovacova, et al., 2016). To increase the number of genomic loci and the sequences per locus, the same two parental individuals were whole‐genome sequenced (WGS) at high depth of coverage (male parent: 37×; female parent: 42×). RAD and WGS raw reads were quality controlled using trimmomatic v0.36 and aligned to the *Am. citrinellus* genome using the program bwa‐mem v0.7.15 (Li & Durbin, 2009) with default parameters. To remove potential duplicate reads, the mapping files of the WGS parental individuals were processed by the *MarkDuplicates* module implemented in the picardtools v1.141 package (http://broadinstitute.github.io/picard/). Then, we used freebayes v1.1.0 (Garrison & Marth, 2012) to infer individuals’ genotypes at polymorphic loci. To obtain a set of reliable markers to be used in downstream linkage map construction and QTL mapping, we used a custom Python script. Briefly, markers where parents were homozygous for different alleles, or heterozygous at only one parent, and where frequencies of genotypes in the F_2_ progeny followed the expected segregation ratio (*χ* ^2^ test threshold *p* = 0.001), were exported. Individuals with more than 10% missing genotypes were removed as were loci present in <80% of individuals. Linkage map construction was performed using joinmap v4.0 (Van Ooijen, 2006) with the regression‐based algorithm implementing the Kosambi mapping function.

QTL mapping was performed independently on body shape and pharyngeal jaw morphology; both traits were measured using geometric morphometric techniques as described in Fruciano, Franchini, Kovacova, et al. (2016). In sum, we mapped three sets of phenotypic data, body shape, pharyngeal jaw shape and their covariation (PLS scores), using a custom R script. First, genotype probabilities were calculated at 0.5‐cM intervals. A multivariate linear model was fit between phenotype and genotype probabilities using Haley‐Knott regression (Haley & Knott, 1992). Then a parametric *p*‐value at each position was obtained and then transformed (−log_10_) to a LOD‐score equivalent (Maga et al., 2015). Significance was assessed through 1,000 permutations by using the empirical distribution of highest LOD score equivalent obtained through permutations (Churchill & Doerge, 1994). For genome‐wide significance, the largest LOD score was used. For chromosome‐level significance, we used the largest LOD score for each linkage group. For each QTL deemed significant at the chromosome level, we computed Bayesian credibility intervals using R/qtl (Broman et al., 2003).

Finally, the identified QTL regions were investigated for potential genomic differences between *Am. citrinellus* and *Ar. centrarchus*. Specifically, we examined whether QTL regions (a) exhibited higher values of both Tajima's D and theta π and whether this effect was only present, or at least stronger, in Midas cichlids (the radiating lineage) than in *Ar. centrarchus*; (b) contained more genes under positive selection; (c) were different in their TE content compared to non‐QTL regions.

## 3 RESULTS

### 3.1 *Amphilophus citrinellus* and *Archocentrus centrarchus de novo* genome assemblies

The contigs of *Amphilophus citrinellus* were de novo assembled using 1,345 million (M) high‐quality paired‐end short reads, totalling 149,920 million base pair (Mbp) (176× coverage), and then scaffolded using both paired‐end and mate‐pair (131 M filtered reads; 5,474 Mbp; 6.4×) information. Dovetail Genomics (Chicago approach) scaffolding technology was then applied to further improve genome contiguity. Using a similar workflow and algorithms, the contigs of *Archocentrus centrarchus* were de novo assembled using 2,130 M paired‐end short reads (297,605 Mbp; 341×) and scaffolded with paired‐end and mate‐pair (101 M filtered reads; 9,017 Mbp; 10.3×) information (Table S1). For the final scaffolding step, we relied on high‐coverage Bionano Genomics optical maps. After assembly, the total length of the *Am. citrinellus* genome was determined to be 854 Mb (scaffold N50: 3.98 Mb), while the *Ar. centrarchus* genome was estimated to be 872 Mb (scaffold N50: 2.76 Mb) (Table S3). *Am. citrinellus* and *Ar. centrarchus* genome assemblies showed high levels of completeness, as revealed by CEGMA and BUSCO approaches that identified >95% complete core vertebrate genes (CVG) and >98% ‘complete + partial’ CVG in either genome (Table S3). Using RNA‐Seq data and homology‐based methods, we annotated 22,557 and 23,639 protein‐coding genes in *Am. citrinellus* and *Ar. centrarchus*, respectively (Table S4). BUSCO metrics showed high completeness in the gene set predicted by our annotation pipeline (>97% complete and >98% ‘complete + partial’ CVG for *Am. citrinellus*; >96% complete and >98 ‘complete + partial’ CVG for *Ar. centrarchus*) (Table S4). The repetitive content of the two genomes, annotated using both custom, species‐specific and publicly available repeat libraries, was 246 Mb for *Am. citrinellus* and 236 Mb for *Ar. centrarchus* (28.81% and 27.08% of the total genome size) (Table S5).

The k‐mer‐based haploid genome size of *Am. citrinellus* and *Ar. centrarchus*, given as the average of the three k‐mer values used (21, 25 and 29), was 754 and 708 Mb, respectively. The unique and repetitive portions of the genome were 688 and 66 Mb for *Am. citrinellus* and 664 and 44 Mb for *Ar. centrarchus*. Heterozygosity was estimated to be 0.289% (*Am. citrinellus*) and 0.334% (*Ar. centrarchus*). The full set of estimated parameters is reported in Table S6, while the frequency distribution of the K‐mer, with the four binomial peaks, is graphically shown in Figure S2.

### 3.2 Demographic history of the *Amphilophus citrinellus* complex and *Archocentrus centrarchus* colonization of crater Lake Xiloá

**FIGURE 1:**
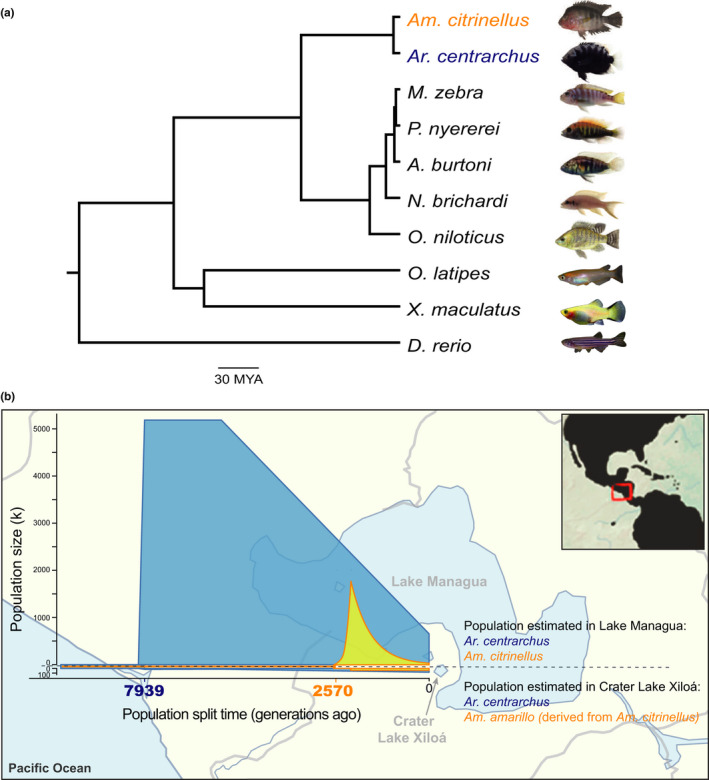
Colonization history of crater Lake Xiloá by *Amphilophus citrinellus* and *Archocentrus centrarchus*. (a) Phylogenetic tree of *Am. citrinellus* and *Ar. centrarchus*, as well as five African cichlids and three other teleost fishes. The phylogeny was constructed using 1,788 one‐to‐one orthologous transcripts; all nodes are supported by 100% bootstrap values. (b) Demographic inference of crater Lake Xiloá colonization by *Am. citrinellus* and *Ar. centrarchus*. The results suggest that Lake Xiloá was colonized 7,939 generations ago by *Ar. centrarchus* and 2,570 generations ago by *Am. citrinellus* [Colour figure can be viewed at wileyonlinelibrary.com]

To examine whether ecological opportunity spurred by the absence of other cichlids (i.e. a priority effect) could have favoured Midas cichlid speciation compared to *Ar. centrarchus* in this crater lake, we used whole‐genome resequencing data of 40 individuals (20 individuals per species/lake) to estimate the colonization history of *Am. citrinellus* and *Ar. centrarchus* inhabiting crater Lake Xiloá. Exploratory demographic analyses with gadma showed that the best fit models support *Am. citrinellus* colonizing Lake Xiloá only 2,570 generations ago, whereas *Ar. centrarchus* is estimated to have colonized Lake Xiloá first, 7,939 generations ago (Figure 1b). A more detailed examination of the demographic history incorporating secondary admixture after the initial crater lake colonization using fastsimcoal confirmed an earlier arrival of *Ar. centrarchus* (5,130 generations ago) as compared to *Am. citrinellus* (2,376 generations ago) in Lake Xiloá, but also revealed evidence for secondary admixture in both lineages (Table S7). Notably, *Ar. centrarchus* showed a higher admixture proportion from the source lake as compared to *Am. citrinellus* (0.524 at 2,840 generations ago vs. 0.352 at 1,478 generations ago). Shorter generation time in *Ar. centrarchus* could explain the differences in colonization times between these two lineages. However, the generation times of *Am. citrinellus* and *Am. amarillo* are similar to the generation time of *Ar. centrarchus* in the lab, as they are both sexually mature at approximately 1 year of age (pers. obs.). Our results, therefore, do not support that Midas cichlids have colonized Lake Xiloá prior to *Ar. centrarchus*.

### 3.3 Transposable elements (TEs) in the genomes of *Amphilophus citrinellus* and *Archocentrus centrarchus*

**FIGURE 2:**
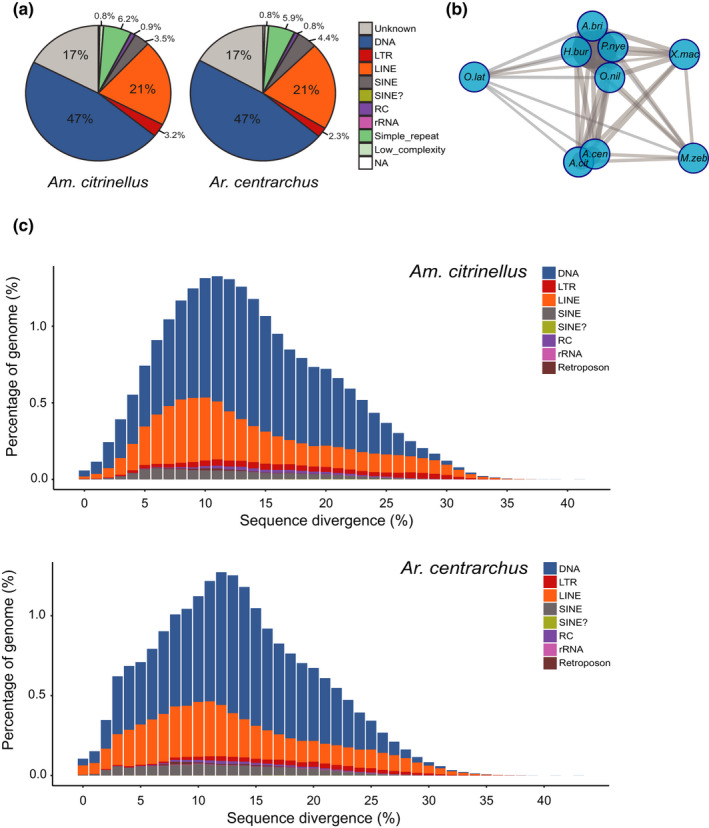
Transposable elements (TEs) characterization and history. (a) Composition of TE families in the genomes of *Amphilophus citrinellus* and *Archocentrus centrarchus*. (b) Euclidean distance network of TE composition in the nine focal ingroup fish genomes (*M.zeb*: *Maylandia zebra*, *P.nye*: *Pundamilia nyererei*, *A.bur*: *Astatotilapia burtoni*, *N.bri*: *Neolamprologus brichardi*, *O.nil*: *Oreochromis niloticus*, *O.lat*: *Oryzias latipes*, *X.mac*: *Xiphophorus maculatus*). The distance between blue circles reflects the differentiation of TE composition between corresponding genomes. (c) Histogram showing TE sequence divergence in the genomes of *Am. citrinellus* and *Ar. centrarchus*. One main peak of TE burst was found in both species [Colour figure can be viewed at wileyonlinelibrary.com]

Transposable elements could have rapidly spread across the Midas cichlid genome following the divergence from its shared common ancestor with *Ar. centrarchus* and thereby could have contributed significantly to the functional diversification and evolution of Midas cichlid gene and genomic architecture (Bohne et al., 2008; Feschotte, 2008). We characterized 246 Mb and 236 Mb TEs in the genomes of *Am. citrinellus* and *Ar. centrarchus*, comprising 28.81% and 27.08% of the genomes, respectively (Table S5). We found relatively similar TE composition in *Am. citrinellus* and *Ar. centrarchus*, where most frequent TE classes are *DNA* transposons, *long interspersed nuclear* elements (LINEs), and un‐characterized (*Unknown*) TEs (Figure 2a). We calculated the Euclidean distance of the composition of each TE and compared it among seven complete cichlid genomes. The distance network reflects the general phylogenetic pattern, where African and Neotropical cichlids form two distinct groups apart from *Maylandia zebra* which does not cluster with the other African cichlids (Figure 2b). Only one main peak of TE burst at 11–12% sequence divergence was found in *Am. citrinellus* and *Ar. centrarchus*, while two peaks of TE burst were consistently identified in each African cichlid species (Figures 2c and S3). The existence of a single TE burst peak with highly similar sequence divergence shared between *Am. citrinellus* and *Ar. centrarchus* shows that neither lineage has experienced a large‐scale lineage‐specific burst of TE, suggesting that this potential genomic mechanism is not involved in the differences in rates of speciation.

### 3.4 Synteny between *Amphilophus citrinellus* and *Archocentrus centrarchus*

We also analysed the synteny and collinearity between the *Am. citrinellus* and *Ar. centrarchus* genomes and detected 1,071 conserved synteny blocks between these two genomes, with a mean length of 996 kb. The genomes exhibit substantial synteny (Figure S4). However, 225 synteny blocks found within the *Am. citrinellus* genome and 75 synteny blocks identified within *Ar. centrarchus* genomes are indicative of potential duplications (Table S8).

### 3.5 Evolution of protein‐coding genes

**FIGURE 3:**
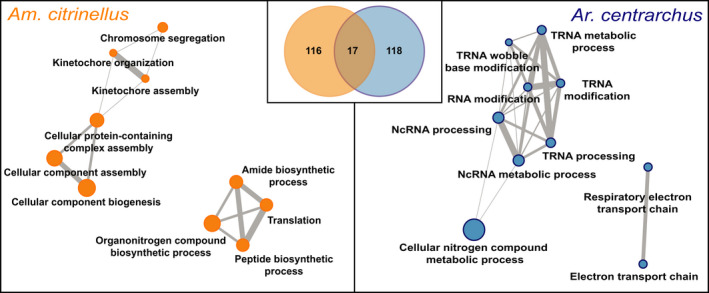
Genes under positive selection. Out of 1,788 single‐copy orthologous genes, we detected positive selection acting on 116 and 118 genes in the genomes of *Amphilophus citrinellus* and *Archocentrus centrarchus*, respectively; 17 genes were found to evolve under positive selection in both species (Venn diagram in the middle). The networks show the functional relationship of significantly enriched gene ontology (GO) terms in the two sets of positively selected genes [Colour figure can be viewed at wileyonlinelibrary.com]

Using the 1,788 single‐copy orthologous genes, we reconstructed the phylogeny of *Am. citrinellus*, *Ar. centrarchus*, five African cichlids and three noncichlid teleost fishes. The divergence time between *Am. citrinellus* and *Ar. centrarchus* was calibrated to 4.8 million years ago using the fourfold degenerated sites from orthologous genes (Figure 1a). To test for positive selection on protein‐coding loci, we included five African cichlids and three noncichlid teleost fishes in this analysis (Figure 1a). In two independent analyses, 1,788 single‐copy ortholog genes were tested for positive selection in *Am. citrinellus* and *Ar. centrarchus*. In this way, we tested whether different selection pressures could have shaped different genes in the two lineages during their independent evolutionary path (i.e. the lineages diverged 4.8 mya). In total, 133 genes were found to be under positive selection in *Am. citrinellus* and 135 genes in *Ar. centrarchus* (Table S9). The two gene lists overlapped only slightly, as only 17 genes were found to evolve under positive selection in both *Am. citrinellus* and *Ar. centrarchus*. The 133 genes under positive selection in *Am. citrinellus* were enriched in Gene Ontology (GO) terms associated with kinetochore, cellular component biogenesis, peptide biosynthesis and ribosome assembly. For *Ar. centrarchus*, beside the GO terms for kinetochore also enriched in *Am. citrinellus*, a different set of enriched GO terms was associated with electron transport, tRNA processing and ncRNA processing (Figure 3; Table S10a,b). No conspicuous gene functional enrichment was detected in the 17 genes under positive selection in both species.

### 3.6 Evolution of gene families

To evaluate the changes in the size of gene families across the phylogenetic tree including the ten fish species (Figure 1a), likelihood estimation was performed for each gene family and branch, assuming the same probability of gene gain and loss. In total, we identified 248 rapidly evolving families in *Ar. centrarchus*, where expansion substantially outnumbered contraction events (217 expanded vs. 31 contracted gene families). A smaller number of rapidly evolving families was identified in *Am. citrinellus*, including 104 expanded and 52 contracted gene families (Table S11; Figure S5).

### 3.7 QTL mapping of ecological traits

**FIGURE 4:**
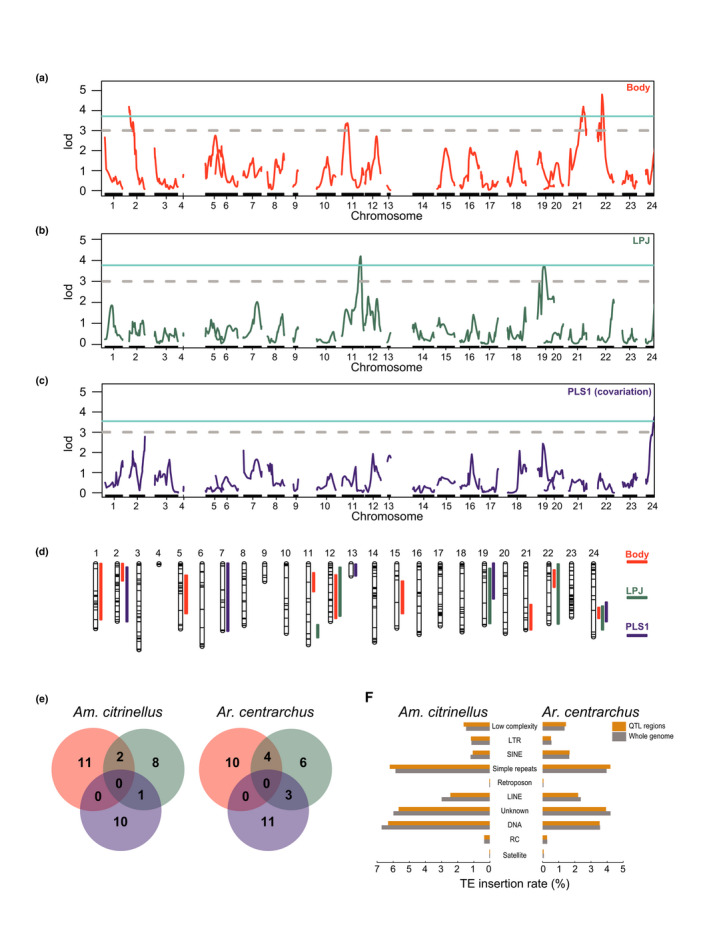
QTL mapping. (a) Genome‐wide LOD scores for each position obtained by mapping body shape (BS). (b) LOD scores of lower pharyngeal jaw morphology (LPJ). (c) LOD scores of partial least square (PLS) scores (covariation of body shape and pharyngeal jaw morphology). (d) QTL intervals of body shape, pharyngeal jaw morphology and their covariation at chromosomal‐level significance in each linkage group. (e) Positively selected genes located in QTL regions. (f) Transposable elements (TEs) changes between *Amphilophus citrinellus* and *Archocentrus centrarchus* within QTL regions [Colour figure can be viewed at wileyonlinelibrary.com]

QTL mapping was conducted on two phenotypic traits, body shape (BS) and lower pharyngeal jaw (LPJ) morphology using a laboratory genetic cross established from a benthic (*Am. astorquii*) and a limnetic (*Am. zaliosus*) species from Lake Apoyo (Fruciano, Franchini, Kovacova, et al., 2016). For mapping, a linkage map was constructed using an F_2_ intercross mapping design and using the *Am. citrinellus* genome as the reference to align the short reads of the two parents and the 306 F_2_ progeny. The map included 527 markers that resolved 24 linkage groups. For BS, three QTL exceeded the genome‐wide threshold (variance explained, VE: 3.01%, 2.03% and 1.46%), while only one QTL was identified for LPJ morphology at the genome‐wide level (VE: 3.58%). One QTL was also found at the genome‐wide level when we mapped covariation between BS and LPJ morphology (VE: 7.04%) (Figure 4a–c). Using less stringent chromosome‐level LOD thresholds, we identified nine QTL for BS, five QTL for LPJ and five QTL for their covariation (Table S12; Figure 4d).

We tested whether theta π and Tajima's D values between QTL and non‐QTL regions differed in each source population. In both lineages, the values obtained were very similar in QTL and non‐QTL regions (Figure S6), the former showing a slightly lower nucleotide diversity (theta π) – which probably reflects the higher gene density of QTL regions (Table S13) – and slightly higher Tajima's D estimates. Overall, no striking patterns were observed within and, most importantly, between species. We then examined if there were more genetic loci under potential selection between *Am. citrinellus* and *Ar. centrarchus* within the identified QTL regions at chromosome‐level thresholds. There were 32 positively selected genes in *Am. citrinellus* falling in QTL regions and 101 positively selected genes located outside the QTL regions. For *Ar. centrarchus*, a very similar pattern was found with 34 out of 135 positively selected genes located in QTL regions. The proportion of genes under selection in the QTL regions is, therefore, similar (*p* = 0.83, chi‐square test; Figure 4e; Table S14). TE changes between *Am. citrinellus* and *Ar. centrarchus* were counted for each synteny blocks. We found 156,091 TE change sites between *Am. citrinellus* and *Ar. centrarchus* out of 365,207 TE sites in QTL regions. The differences in TE loci were slightly lower in QTL regions compared to the rest of the genome (*p* < 0.001, chi‐square test; Table S15). However, the changes in ‘low complexity’ and ‘simple repeats’ categories were relatively more elevated in QTL regions (Figure 4f).

## 4 DISCUSSION

Our comparative analyses have provided several potent insights into the adaptive radiation of Nicaraguan crater lake cichlids. The inferred earlier colonization of crater Lake Xiloá by *Ar. centrarchus* (the nonradiating lineage) argues against a role for priority effects in promoting diversification in Midas cichlids (the radiating lineage) (Figure 1). This result is consistent with previous work on Midas cichlids and *Ar. centrarchus* based on lower‐resolution genetic data (Franchini et al., 2017; Fruciano, Franchini, Kovacova, et al., 2016; Fruciano, Franchini, Raffini, et al., 2016). Our further examination of demographics reveals a second wave of colonization for both species, which is also consistent with previous analysis in *Ar. centrarchus* (Franchini et al., 2017). However, the predicted secondary invasion events do not invalidate our inference here that *Ar. centrarchus* likely occupied crater Lake Xiloá before Midas cichlids began radiating. The priority of colonization alone is unlikely to provide a reasonable explanation for the conditions favouring the adaptive radiation of Midas cichlids. Nonetheless, we cannot exclude that the higher admixture proportion shown by *Ar. centrarchus* from the source lake, when compared to *Am. citrinellus*, has played a role in preventing its diversification. In this scenario, the high level of gene flow from Lake Managua could have been one of the mechanisms contributing to genetic and morphological homogenization in the possibly ongoing process of *Ar. centrarchus* radiating in crater Lake Xiloá.

To investigate which aspects of genomic architecture potentially played a role in the adaptive radiation of Midas cichlids, we first comparatively examined several genome‐wide characteristics of the newly assembled *Am. citrinellus* and *Ar. centrarchus* genomes. The fully sequenced reference genomes of these two species provided several insights into which genomic features differ between these two lineages, and which do not. The genome assembly size of *Am. citrinellus* was determined to be 854 Mb and for *Ar. centrarchus* was 872 Mb. The similarity in genome sizes suggests there have not been radical changes in the overall structure of the genome between these two species. Both of these lineages are also known to show conservation of 24 haploid chromosomes (Thompson, 1979). The lack of major chromosomal gain or losses is further supported by our finding of highly conserved synteny between the two lineages. This conservation coupled with our genome size estimates suggests that major changes in genome size or chromosome structure are not responsible for the differential tendency of the two lineages to adaptively radiate. This is particularly relevant in light of the fact that chromosomal rearrangements could affect the linkage of QTL for distinct adaptive traits which, in turn, has been suggested as a potential facilitator of sympatric speciation in Midas cichlids (Fruciano, Franchini, Kovacova, et al., 2016). These putative rearrangements could have affected the regions of overlap between QTL regions for distinct traits. However, we note that confidence intervals for QTL regions identified here are broad. Thus, we could only test whether major chromosomal rearrangements were occurring, and cannot discount those smaller rearrangements, shown by synteny breaks between two species, at a local scale to play a role for the different propensity of the two lineages to speciate.

Genome‐wide abundance of transposable elements (TEs) could represent another major factor affecting the possibility for adaptive radiations to unfold (Niu et al., 2019; Serrato‐Capuchina & Matute, 2018). In cichlids, TE composition can change rapidly within a very short evolutionary period (Figure 2b). However, we found that genome‐wide TE composition is similar between *Am. citrinellus* and *Ar. centrarchus* (Figure 2a,b), and there is no evidence for a lineage‐specific genome‐wide TE expansion event following the ancestral split between *Am. citrinellus* and *Ar. centrarchus*. Our results cannot discount, however, the possibility that a few TEs, as opposed to genome‐wide variation in TE number and composition, could have had a disproportionate effect on the Midas cichlid adaptive radiation. Further, we found no evidence for the hypothesis that a global expansion in gene families is responsible for the differential propensity of these cichlid lineages to radiate. One might have expected that Midas cichlids show an expansion in gene families creating a ‘substrate’ for evolution to act upon and contribute to their disproportionate phenotypic diversity. If anything, the results support the opposite pattern as *Ar. centrarchus* has a larger number of gene families that underwent expansion. However, these genome‐wide patterns could mask the expansion of a few gene families that had a disproportionate effect on the propensity of Midas cichlids to radiate.

Assuming that different genes were positively selected in the two lineages during their 4.8 million years of independent evolution and that these could be related to their different adaptive potential, we examined the evolution of protein‐coding genes in each lineage (Figure 3). Genome‐wide examination of positive selection on coding regions does reveal interesting patterns of disparate signatures of selection between *Am. citrinellus* and *Ar. centrarchus*. For instance, we found signals of positive selection on genes related to regulatory processes in both *Am. citrinellus* and *Ar. centrarchus*, but the positively selected genes in each lineage were enriched for different pathways. Positively selected genes in *Ar. centrarchus* showed enrichment in transfer RNAs (tRNAs) and noncoding RNAs (ncRNAs) while *Am. citrinellus* was enriched in ribosomal and peptide synthesis pathways. Ribosomal genes are known to have critical functions in gene regulation (Barna, 2017). Also, ribosomal genes in cichlids have particularly long 3′UTRs and more novel miRNA target sites compared to other fishes (Xiong et al., 2018). Protein‐coding changes coupled with the previously detected noncoding modifications of these ‘meta‐regulators’ could have provided more regulatory possibilities during the Midas cichlid adaptive radiation (Franchini et al., 2016; Xiong et al., 2018, 2019).

We also considered whether genomic characteristics in regions containing phenotypically important QTL recovered from the crosses of crater lake Midas cichlids could explain their propensity to radiate. In particular, we performed QTL mapping using our new de novo genome of *Am. citrinellus* as the reference and incorporated previously published genetic and phenotypic information on the Midas cichlid radiation present in Lake Apoyo (Fruciano, Franchini, Kovacova, et al., 2016). We re‐examined the QTL of body shape and pharyngeal jaw shape, as these traits have repeatedly diverged in Midas cichlid crater lake radiations (Franchini et al., 2014; Fruciano, Franchini, Kovacova, et al., 2016; Kautt et al., 2018). By using the *Am. citrinellus* genome as a reference and chromosomal‐level significance thresholds, we confirmed a number of previously known and also identified several new QTL regions (Table S13) compared to our previous study (Fruciano, Franchini, Kovacova, et al., 2016). These focal genomic regions were then scanned for broad‐scale patterns in TEs and genes under selection. However, we found no substantial difference between the two species in these regions. Future work could identify additional QTL regions containing adaptive traits in Midas cichlids as well as in *Ar. centrarchus*, making these regions available for scans of TEs and genes under selection. However, considering the patterns we document for both genome‐wide and at putative QTL regions, it is unlikely that new QTL regions will display especially high variation in TE content or an elevated number of genes under selection. The theta π and Tajima's D statistics computed for both Midas cichlids and *Ar. centrarchus* also did not reveal any clear difference between the two lineages. We did detect variation between QTL regions and the rest of the genome with lower theta π and higher Tajima's D in QTL regions. However, the lower theta π may be due to higher gene density in QTL regions. The higher Tajima's D would be in principle consistent with the hypothesis that QTL regions also experience increased balanced selection in the source populations. However, the differences we observed in Tajima's D across genomic region types – while remarkably consistent across multiple species/lakes – are very subtle and do not allow informed speculation with respect to the causes of this pattern. Most importantly for the purpose of this study, the pattern of higher Tajima's D in QTL regions is the same whether we analyse Midas cichlids or *Ar. centrarchus*, and therefore does not seem to explain the disparity in the propensity to radiate across the two lineages. Clearly, comparisons of QTL regions with the rest of the genome have limitations due to the relatively large confidence intervals for QTL regions. These large intervals are due to relatively small sample sizes for a QTL study, as well as to the F_2_ design of the mapping population that is dictated by the long generation time and tank space demands of Midas cichlids. At the same time, likely, several QTL regions of very small effect were not detected as we expect that our focal traits are highly polygenic. This further lowers the power of comparisons of identified QTL regions with the rest of the genome. Yet, all of our analyses here lead to the conclusion that methods such as QTL mapping that facilitate the narrowing of the genomic intervals responsible for phenotypic divergence will most profitably illuminate the genomic mechanisms responsible for adaptive radiations like the Midas cichlids.

Pinpointing the mechanisms responsible for promoting adaptive radiation will continue to demand an integrated examination of biological mechanisms. Using a combination of two de novo genome assemblies, population‐level whole‐genome resequencing and phenotypic data, we attempted to address these mechanisms in an iconic group of sympatrically speciating, rapidly radiating cichlid fish as well as a closely related, nonradiating species. We were able to reject the hypothesis that ecological opportunity (i.e. Midas cichlids colonizing the crater lake environment first and adaptively radiating at the expense of newcomers) played a substantial role in one of the most diverse subsets of the Midas cichlid adaptive radiation. Further, and perhaps most importantly, we rejected several hypotheses that genome‐wide characteristics like TE content, broad chromosomal rearrangements, the number of positively selected genes, differences in rates of molecular evolution, or the expansion of gene families may have fuelled the radiation of Midas cichlids. Clearly, several other factors may explain the disparity in diversity between Midas cichlids and closely related cichlids inhabiting the same lakes. Phenotypically, differences in the degree and type of sexual dimorphism, in the complexity of sexual signals and in the ability to respond plastically in the initial phases of colonization of new environments could all play a role (West‐Eberhard, 2003). The role of the genetic architecture of traits under selection could also have greatly influenced the Midas cichlid radiation (Kautt et al., 2020). Among several possible genomic factors, we have here identified positive selection on different major gene functions as a promising candidate for determining the propensity of Midas cichlids to radiate. Positive selection on several loci including those contributing to ribosome assembly could have provided the molecular pre‐adaptation that allowed Midas cichlids to explosively diversify. Overall, our results point to a disproportionate role of genomically localized, rather than global, genomic factors in facilitating the propensity of these fishes to radiate.

## AUTHOR CONTRIBUTIONS

PX, CDH, CF, WYW, AN, AFK, OS, MP and PF performed the bioinformatic analyses. PF, CDH, CF, SK and AM collected the material and conceived the study. PX, CDH and PF wrote the manuscript with input from all authors.

## ETHICAL APPROVAL

The authors declare no competing interests.

## Supporting information

Fig S1‐S6

Table S1‐S15

## Data Availability

Raw Illumina sequences were deposited in the NCBI's Sequence Read Archive (SRA): de novo genome assembly (PRJNA682377); whole‐genome resequencing (PRJEB38173, PRJNA682530); transcriptome assembly (PRJNA544930; PRJNA635556, PRJNA516733). The assembled genomes and transcriptomes of *Amphilophus citrinellus* and *Archocentrus centrarchus*, and the Bionano optical map generated for *Ar. centrarchus* can be downloaded from Dryad (https://doi.org/10.5061/dryad.gmsbcc2m0).
