# Supplementary material for: The comparative genomic landscape of adaptive radiation in crater lake cichlid fishes
Source: Mol Ecol. 2021 Jan 6;30(4):955–72. doi: 10.1111/mec.15774 (PMC8607476; doi:10.1111/mec.15774)
Supplement: Supplementary file 1 — Fig S1‐S6 [file MEC-30-955-s001.docx]

**Supplementary Figures**

**The Comparative Genomic Landscape of Adaptive Radiation in Crater Lake Cichlid Fishes**

Peiwen Xiong^1^, C. Darrin Hulsey^1,2^, Carmelo Fruciano^1,3^, Wai Y. Wong^4^, Alexander Nater^1^, Andreas F. Kautt^1,5^, Oleg Simakov^4^, Martin Pippel^6^, Shigehiro Kuraku^7^, Axel Meyer^1,*^, Paolo Franchini^1,*^

^1^Department of Biology, University of Konstanz, Konstanz, Germany

^2^Current Address: School of Biology and Environmental Science, University College Dublin, Dublin, Ireland

^3^National Research Council (CNR) – IRBIM, Messina, Italy

^4^Department of Molecular Evolution and Development, University of Vienna, Vienna, Austria

^5^Current Address: Department of Organismic and Evolutionary Biology, Harvard University, Cambridge, MA, USA

^6^Max Planck Institute of Molecular Cell Biology and Genetics, Dresden, Germany

^7^Laboratory for Phyloinformatics, RIKEN Center for Biosystems Dynamics Research (BDR), Kobe, Japan

^*^Corresponding authors: Paolo Franchini, paolo.franchini@uni-konstanz.de; Axel Meyer, axel.meyer@uni-konstanz.de

*Table of contents*

| Supplementary Figure S1 . . . . . . . . . . . . . . . | 2 |
| --- | --- |
| Supplementary Figure S2 . . . . . . . . . . . . . . . | 3 |
| Supplementary Figure S3 . . . . . . . . . . . . . . . | 4 |
| Supplementary Figure S4 . . . . . . . . . . . . . . . | 5 |
| Supplementary Figure S5 . . . . . . . . . . . . . . . | 6 |
| Supplementary Figure S6 . . . . . . . . . . . . . . . | 7 |

**Supplementary Fig. S1**


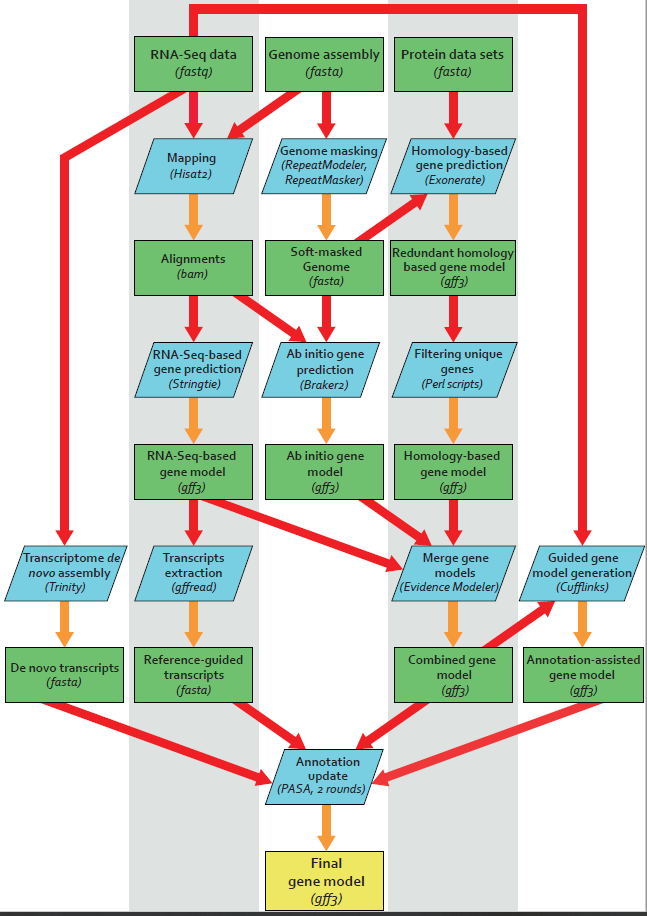


**Supplementary Figure S5.** Schematic overview of the annotation pipeline used to predict protein-coding genes in the genomes of *Amphilophus citrinellus* and *Archocentrus centrarchus*.

**Supplementary Fig. S2**

| 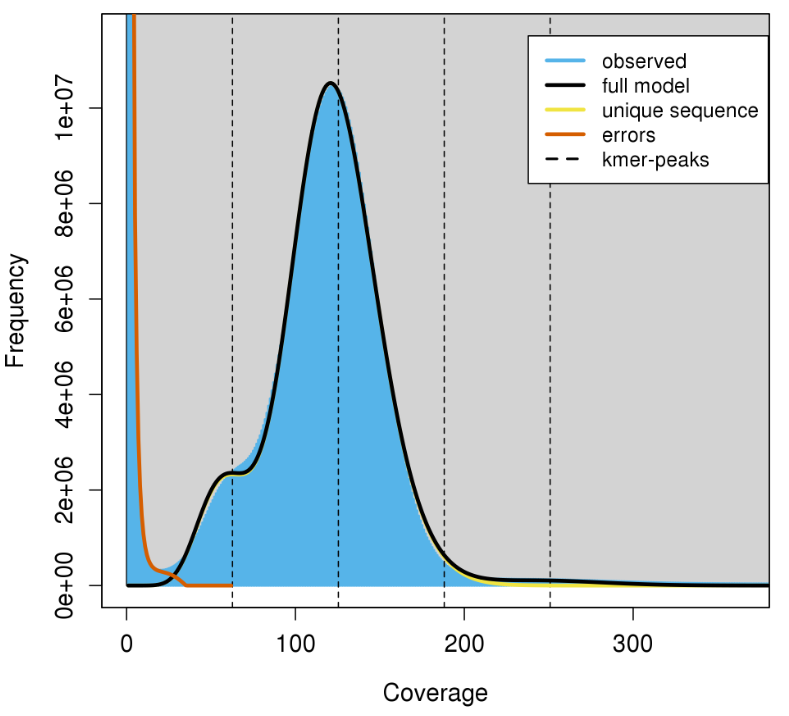 | 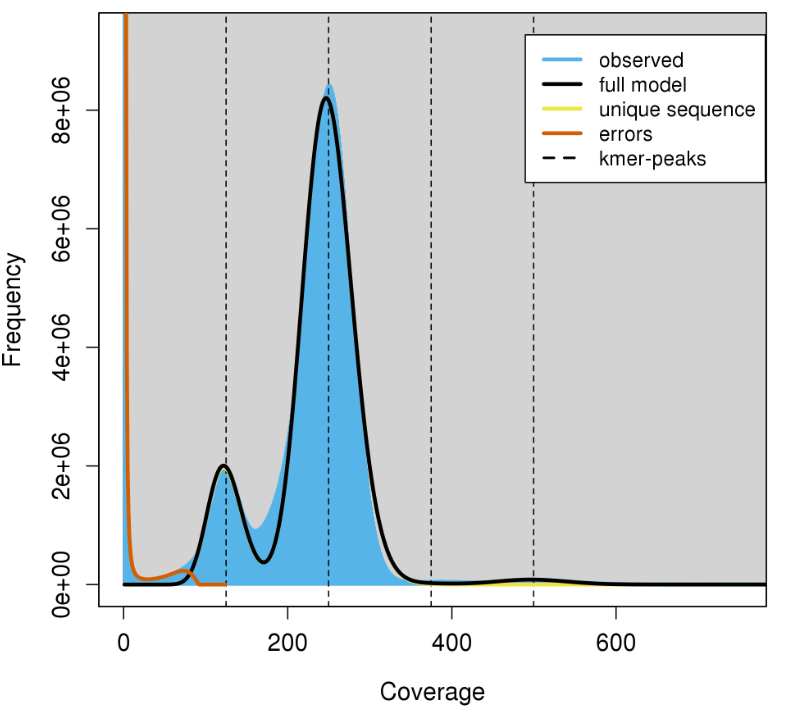 |
| --- | --- |

**Supplementary Figure S2.** GenomeScope k-mer profile plot of the *Amphilophus citrinellus* (left) and *Archocentrus centrarchus* (right) data sets showing the fit of the model (black) to the observed k-mer frequencies (blue). The plots wer built with a k-mer length of 25.

**Supplementary Fig. S3**

**
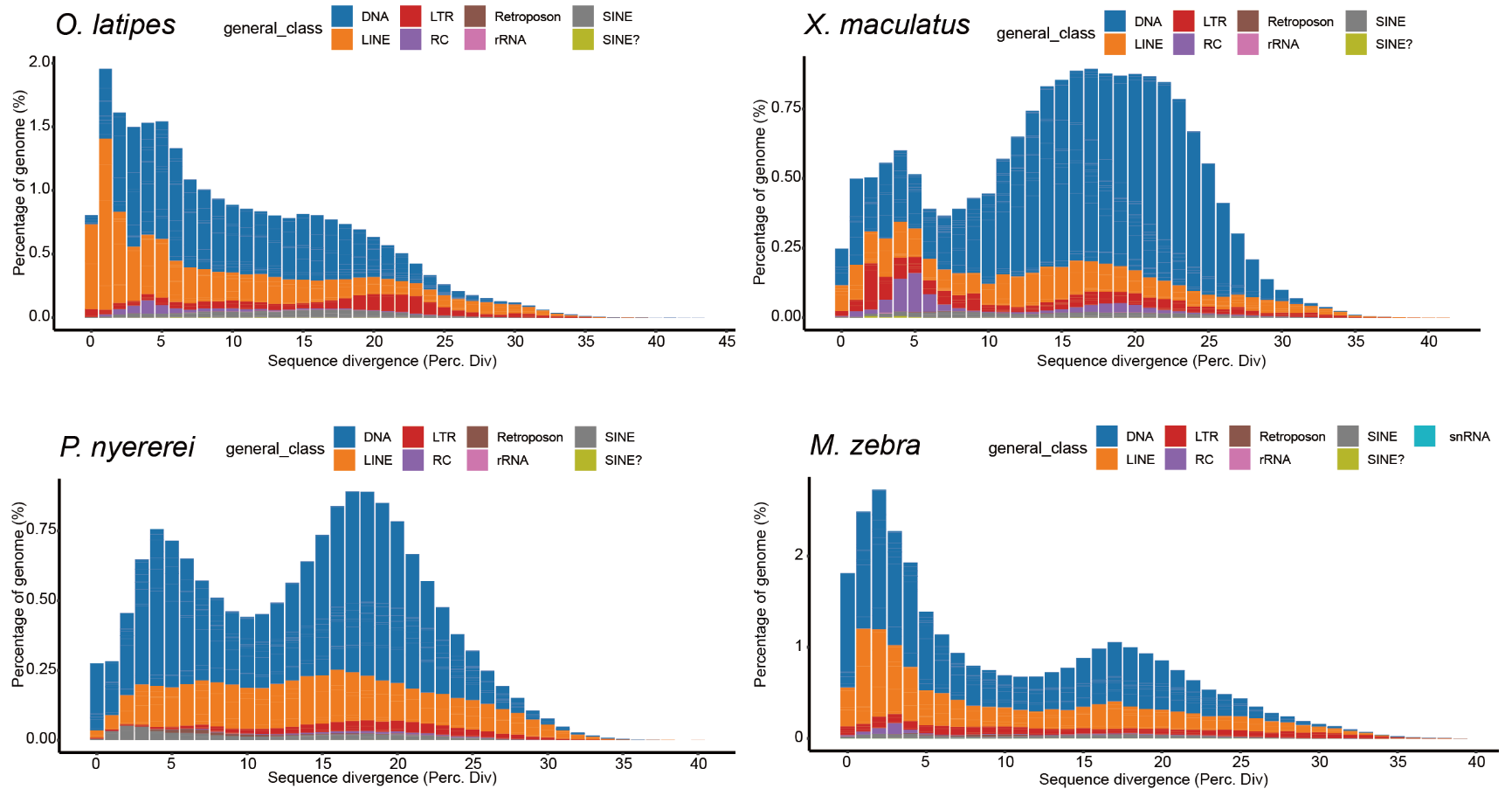
**

**
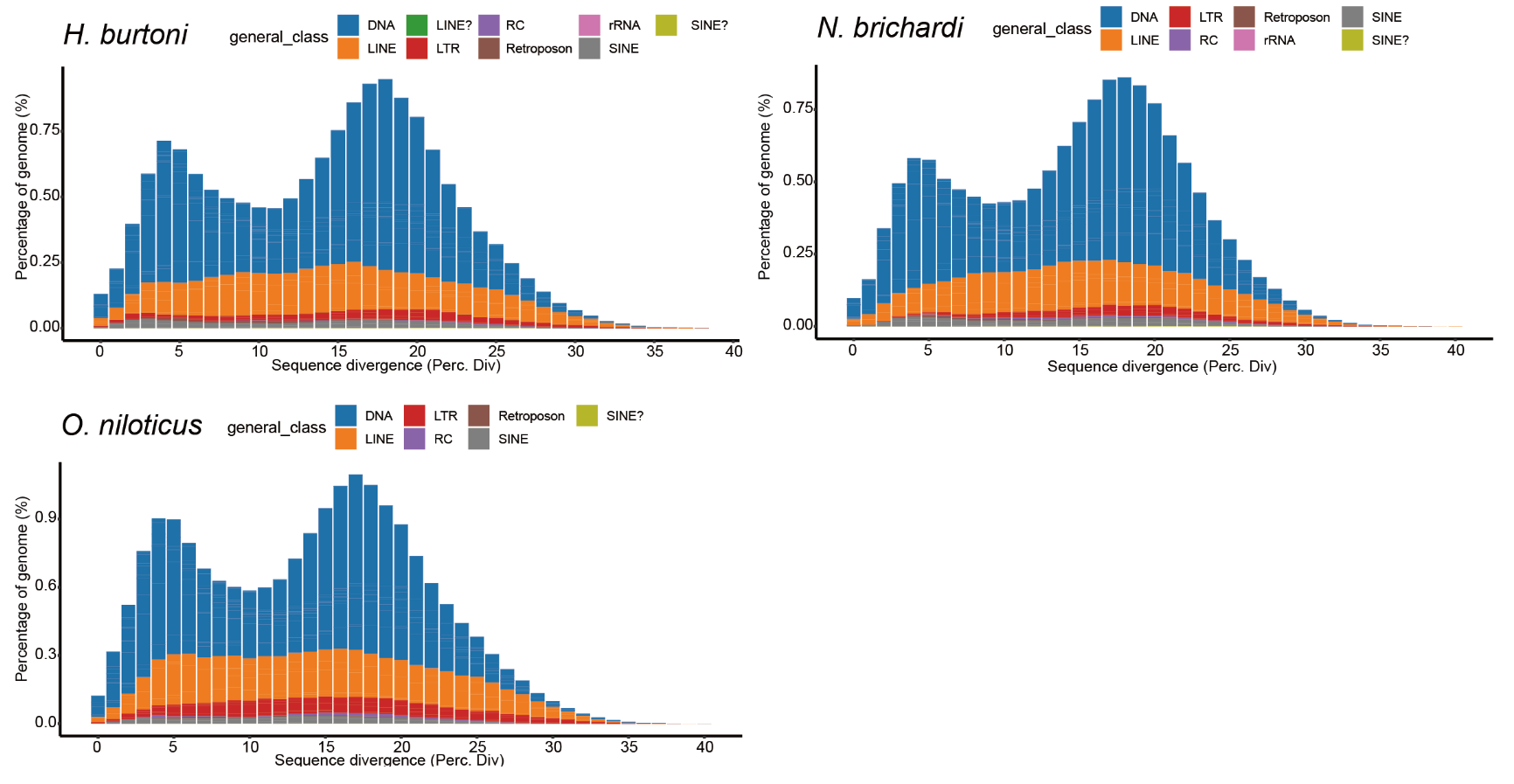
**

**Supplementary Figure S3.** Transposable element (TE) sequence divergence in the genomes of African cichlid fishes (*M. zebra*, *P. nyererei*, *H. burtoni*, *N. brichardi*, *O. niloticus*) and non-cichlid teleost fishes (*O. latipes*, *X. maculatus*).

**Supplementary Fig. S4**


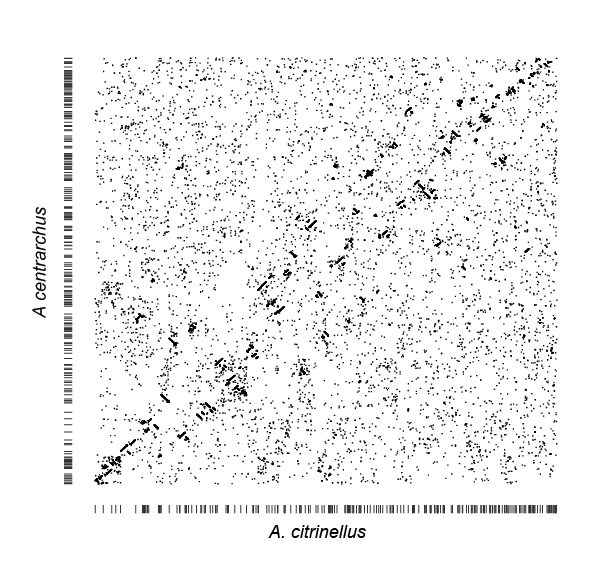


**Supplementary Figure S4.** Macro-synteny plot between the genomes of *Amphilophus citrinellus* and *Archocentrus centrarchus*.

**Supplementary Fig. S5**


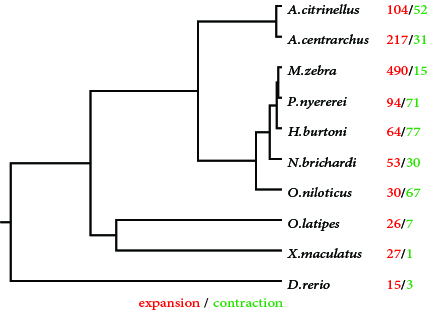


**Supplementary Figure S5.** Significantly expanded and contracted gene families in the ten fish species used in the study.

**Supplementary Fig. S6**


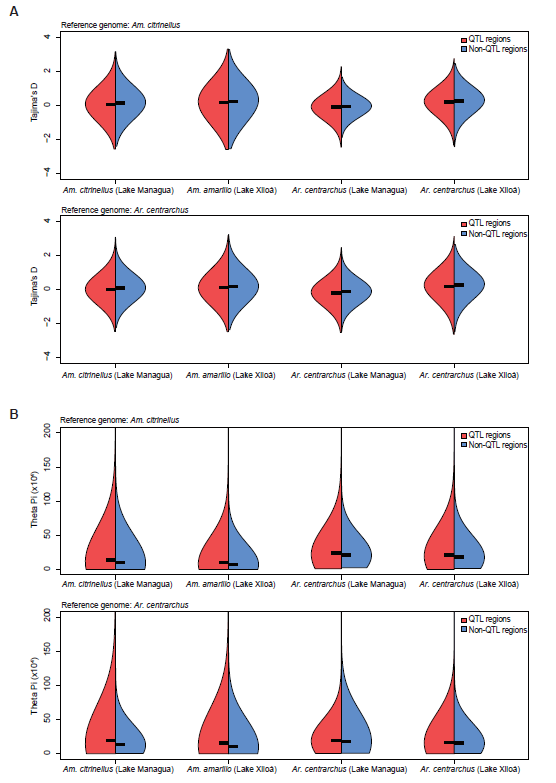


## **Supplementary Figure S6.** **(A)** Tajima’s D and **(B)** theta π values calculated in QTL and non-QTL genomic regions in the populations of *Am. citrinellus* and *Ar. centrarchus*. The estimates were inferred using the population genomic data of each population aligned to both reference genomes.
